# Renal Histologic Findings in Necropsies of Type 2 Diabetes Mellitus Patients

**DOI:** 10.1155/2022/3893853

**Published:** 2022-09-06

**Authors:** Luis D'Marco, María Jesús Puchades, Victor Escudero-Saiz, Elena Giménez-Civera, Liria Terradez, Anais Moscardó, Juan Antonio Carbonell-Asins, Elisa Pérez-Bernat, Isidro Torregrosa, Francesc Moncho, Jorge Navarro, José Luis Górriz

**Affiliations:** ^1^Universidad Cardenal Herrera-CEU, CEU Universities, Valencia, Spain; ^2^Nephrology Department, Hospital Clínico Universitario, INCLIVA, Valencia, Spain; ^3^University of Valencia, Valencia, Spain; ^4^Nephrology Department, Hospital Clinic, Barcelona, Spain; ^5^Pathology Department, Hospital Clínico Universitario, Valencia, Spain; ^6^Bioinformatics and Biostatistics Unit, INCLIVA Biomedical Research Institute, Valencia, Spain; ^7^Hospital Clínico Universitario Valencia, Universidad de Valencia, CIBERESP, Spain

## Abstract

**Background:**

Very few studies have analyzed early histologic lesions of diabetic nephropathy (DN) in patients without signs of clinical involvement (microalbuminuria). In this study, we analyzed renal histologic lesions in necropsies of diabetic patients with or without previous signs of DN.

**Methods:**

Histological material was analyzed from 21 autopsies of type 2 diabetes mellitus (T2DM) patients (9 with albuminuria and 12 without albuminuria) and 4 controls. Histologic lesions were evaluated according to the Tervaert classification.

**Results:**

Kidneys of diabetic patients presented significantly higher scores in most histologic indices analyzed (glomerular basal membrane thickening, mild and severe mesangial expansion, nodular sclerosis, interstitial fibrosis, and tubular atrophy) than in nondiabetic controls (*p* < 0.01 in all cases). In contrast, no significant differences were detected between histologic scores when comparing the 21 diabetic patients with and without albuminuria. A significant percentage of cases without albuminuria showed moderate to severe histologic lesions, particularly severe mesangial expansion and severe glomerular vascular lesions. No significant differences were found in age, blood pressure, diabetes vintage, BMI, HbA1c, cholesterol, triglycerides, or treatments between the two (albuminuric vs. nonalbuminuric) T2DM patient groups.

**Conclusions:**

Our data suggest that histologic lesions of DN are present in the early stages of the disease, even without albuminuria presence. More precise and earlier metabolic control is recommended in T2DM, and monitoring of risk factors can play a role in DN development.

## 1. Introduction

The prevalence of diabetes mellitus (DM) is around 425 million people worldwide, and this figure is predicted to increase to over 600 million by 2045 [1]. Diabetic nephropathy (DN) is the leading cause of morbidity and mortality in patients with DM, and estimations indicate that 30–40% of DM patients will develop DN [2]. In this context, excess all-cause mortality in DM patients is associated with chronic kidney disease (CKD). Beyond its diagnostic and prognostic value, microalbuminuria is used as a marker of endothelial dysfunction, indicating the existence of glomerular compromise. Patients who express microalbuminuria also have a higher risk of progression to proteinuria and end-stage kidney disease (ESKD). This urinary biomarker represents an important variable for assessing DN progression [3]. However, the currently accepted paradigm is that degree of albuminuria/proteinuria alone is not sufficient for prognostic evaluation, since not all patients with type 2 (T2) DM who develop renal failure have albuminuria/proteinuria [4].

Following the Tervaert pathological classification for DN, studies have shown that both glomerular and interstitial involvements are independently associated with CKD [5]. Tubulointerstitial damage is related to glucotoxicity, which accelerates CKD progression and is closely related to renal function decline; indeed, studies point to tubular damage as a better predictor of CKD progression than classic markers such as albuminuria [6]. Of interest, in earlier stages of CKD, the presence or absence of certain pathological findings such as nodular and exudative lesions and/or mesangiolysis is predictive of adverse renal events (dialysis, doubling of serum creatinine, or sustained decrease in estimated glomerular filtration rate [eGFR]). These pathological lesions are relevant in T2DM patients with normal and abnormal eGFR or microalbuminuria, becoming predictors of cardiovascular and adverse renal events [7].

Prevention of DN is the key to avoiding disease progression, and most therapeutic measures have focused particularly on patients with incipient nephropathy (microalbuminuria) or eGFR decrease. Recent KDIGO guideline recommendations on diabetes management are centered on T2DM patients with CKD (urine albumin-to-creatinine ratio >30 mg/g or eGFR <60 mL/min/73 m^2^) [3], yet DN prevention measures should be established in earlier stages before microalbuminuria develops.

Renal biopsy in T2DM patients is usually indicated in those with significant renal manifestations such as severe proteinuria, microscopic hematuria, rapid unexplained worsening of kidney function, or over 30% decline in eGFR after initiating RAAS inhibition [8]. In this regard, some studies have analyzed early DN lesions in patients without clinical signs of this involvement (microalbuminuria) [9]. Nonetheless, although not a routine technique, renal biopsy remains the gold standard of DN diagnosis and recent evidence supports determining renal histologic involvement in this disease. For this reason, very few studies to date have analyzed renal histologic alterations in the early stages of T2DM.

The main aim of the study was to analyze whether T2DM patients without microalbuminuria present histologic lesions of DN. For this purpose, histologic samples of renal tissues were analyzed in necropsies from diabetic patients with or without microalbuminuria, and these findings were compared with renal samples from autopsies of nondiabetic patients as a control group.

## 2. Methods and Material

Samples were selected from autopsies performed at the Hospital Clínico Universitario of Valencia between 2015 and 2017. A total of 25 autopsies were analyzed: 21 in T2DM patients (9 in albuminuric and 12 in nonalbuminuric patients) and 4 nondiabetic matched controls. Histological data were therefore included for all cases according to the work protocol and classification of histologic lesions. Once selected, the data of all these clinical and pathological variables were retrieved from hospital electronic medical records.

### 2.1. Processing of Histologic Material

Histological data were reviewed by one expert pathologist in renal histology who was blinded to the study data. Given that most autopsies show a greater or lesser degree of acute tubular necrosis (ATN), we decided to evaluate only vascular lesions, although analysis of interstitial lesions was also attempted since most autopsies could establish scores for fibrosis with associated tubular atrophy or inflammatory findings.

Renal autopsy histology material was fixed in 10% formalin and embedded in paraffinic tissue. All samples were stained by Dako Cover Stainer (Dako) Autostainer for hematoxylin-eosin (H-E) staining and Artisan Link Pro (Dako) for special stains such as PAS, Masson's trichrome, and Jones stain (silver).

The histologic score was based on the Tervaert classification of the American Society of Pathological Anatomy [5]:

- Glomerular lesions. Total number of glomeruli, the number of glomeruli with thickening of the glomerular basement membrane, the number of glomeruli with the mild expansion of the mesangium, the number of glomeruli with severe expansion, the presence of nodules, the number of glomeruli with sclerosis, and glomeruli with global sclerosis (>50%)
- Tubulointerstitial lesions. Interstitial fibrosis and tubular atrophy (IFTA), subdivided into four points according to the affected percentage: 0 (no fibrosis), 1 (<25%), 2 (25–50%), and 3 (>50%), and the presence of interstitial inflammation and whether or not it is associated with areas of IFTA: 0 (absence of interstitial inflammation), 1 (presence, also associated with areas of IFTA), and 2 (presence, not associated with areas of IFTA)
- Vascular lesions. Arteriolar hyalinosis, arteriosclerosis (establishing two degrees based on the predominant thickness of the intima and media layers between), and arteriolar drops
- Others. Capsular drops, glomerular and tubular adhesions, tubular glomeruli (atrophy of the proximal convoluted tubule (TCP) in the urinary pole), and the presence of urinary cysts and fibrinolysis

### 2.2. Clinical Data Collection

Clinical data were retrieved from hospital electronic records, including the following clinical characteristics: age (years), duration of diabetes until death (months), sex, height (cm), weight (kg), body mass index (BMI) (kg/m^2^), blood pressure (mmHg), analytical data, eGFR (mL/min/1.73 m^2^), previous diagnosis of retinopathy, history of cardiovascular disease (ischemic heart disease, transient ischemic attack (TIA)/stroke, peripheral vascular disease, and heart disease), arterial hypertension (HTN), and drug treatments: antiaggregate, oral anticoagulants, RAAS blockers, and hypoglycemic agents.

### 2.3. Statistical Analysis

The Shapiro-Wilk test was used to determine sample distribution. Given the sample size and distribution, quantitative variables are expressed as a median and interquartile range and qualitative variables as percentages. The Mann-Whitney *U* test was used to compare differences between continuous variables in the two independent groups. Statistical significance was set at *p* < 0.05. For categorical variables, the comparison was made using Fisher's test as counts were too low to use the chi-squared test. Descriptive analysis and the Mann-Whitney *U* test were performed using the IBM SPSS Statistics version 19.0 software, while the R software was used to apply Fisher's exact test. All statistical tests with *p* values below 0.05 were considered significant.

## 3. Results

**Figure 1:**
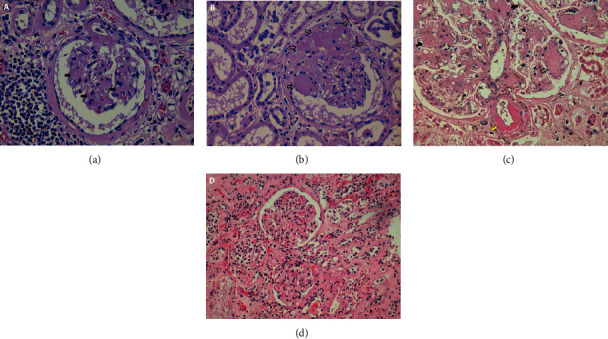
Kidney histological findings. (a) HE 400x, glomeruli with mild mesangial expansion (black arrows) and Bowman's capsule fibrosis. Findings of chronic interstitial fibrosis and tubular atrophy (IFTA) on the left side of the figure, in a nonalbuminuric diabetic subject. (b) HE 400x, nodular lesions (Kimmelstiel-Wilson) (empty arrows) that correspond to a class III: nodular sclerosis in the histologic classification system, in a diabetic albuminuric subject. (c) HE 400x, severe mesangial expansion, Kimmelstiel-Wilson nodules, and hyalinosis of efferent arteriole (yellow arrow) in a diabetic albuminuric subject. (d) HE 200x, glomeruli with nonspecific changes from a sample of the control group.

Clinical and analytical data from autopsies of diabetic patients with and without albuminuria and controls are summarized in Table 1 and Supplemental Table 1 (S1). Albuminuria values in patients with and without this condition differentiated the characteristics of the two groups since those patients without albuminuria showed a very low range (mean 10.53 mg/g with an interquartile range of 3.08–17.69), which contrasted with the much higher ranges observed in the albuminuria patient group (median 223 mg/g with an interquartile range of 153–393). Some histological samples of the groups are shown in Figure 1.

Patients with albuminuria showed higher serum creatinine (*p* = 0.032) levels and lower eGFR (*p* = 0.037) than nonalbuminuric ones. Moreover, the latter group received more insulin and fewer metformin doses (*p* = 0.031 in both cases). No significant differences between groups were found in clinical or analytical data or treatments received (Tables 1 and S1). These data show that the three patient groups (nonalbuminuric, albuminuric, and controls) had similar and therefore comparable clinical characteristics, despite the limited number of patients.

### 3.1. Comparison of Diabetics and Nondiabetic Controls

The patients' clinical and histologic characteristics are shown in Table 2. For the histologic study, we analyzed a large number of glomeruli (median, 146) in each sample analyzed (interquartile range: 112–209).

**Figure 2:**
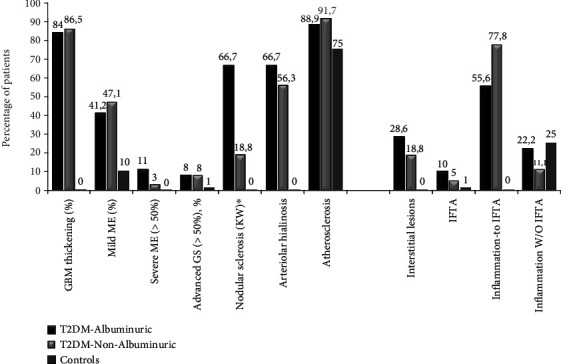
Percentage of patients with different histologic lesions. (a) Glomerular lesions. (b) Interstitial lesions. The number of glomeruli evaluated in each group was albuminuric patients 113 [94–228.5], nonalbuminuric patients 159.5 [120.75–213.5], and controls 129.5 [82.25–186.5]. There were significant differences in all lesions when comparing diabetic patients and nondiabetic controls. No significant differences were found when comparing albuminuric and nonalbuminuric T2DM patients except for the presence of nodular sclerosis∗ (*p* = 0.031).

Comparing findings of diabetic patients with nondiabetic controls, as expected, we observed that patients with diabetes presented significantly higher scores in glomerular basement membrane thickening, mesangial expansion, and arterial hyalinosis (*p* < 0.015 in all cases), all with signs related to glomerular lesions of T2DM. Likewise, diabetic patients had higher scores in interstitial lesions, inflammation, and tubular atrophy than nondiabetic controls (*p* < 0.05). There were no significant between-group differences related to arteriosclerosis lesions in the scores (Figure 2).

### 3.2. Comparison of Diabetic Patients with and without Albuminuria

**Figure 3:**
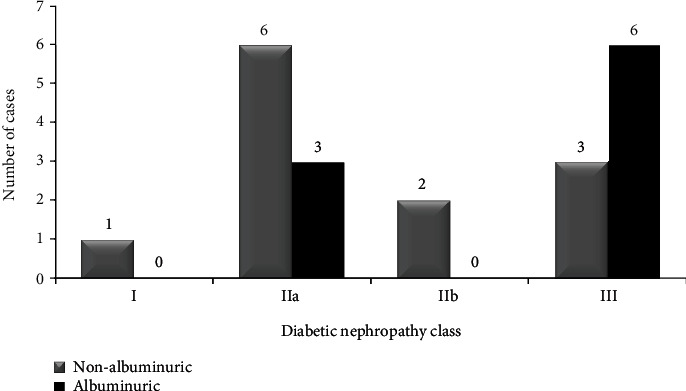
The number of cases in the different classes of glomerular diabetic nephropathy lesions. Glomerular lesions in all diabetic patients. We show albuminuric (black) against nonalbuminuric (gray) (*n* = 21) patients.

**Figure 4:**
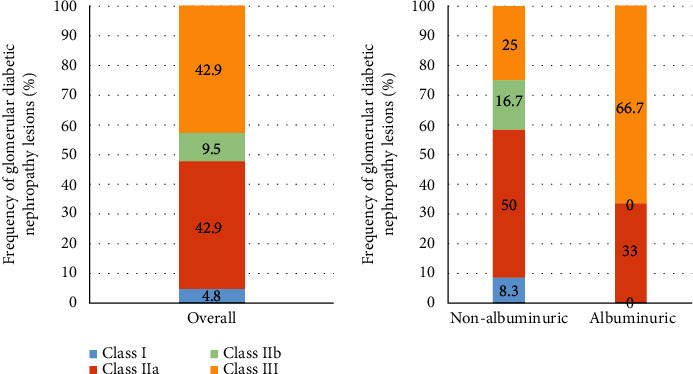
Frequency of different classes of glomerular diabetic nephropathy lesions. Frequency of different classes of glomerular diabetic nephropathy lesions in all diabetic patients (left panel). The right panel shows the different classes according to albuminuric status (*n* = 21).

When histologic lesions were compared between albuminuric and nonalbuminuric patients, we found significant differences regarding the presence of nodular sclerosis (presence of Kimmelstiel-Wilson nodules): 66.7% in albuminuric vs. 18.8% in nonalbuminuric (*p* = 0.031), but none were found for the remaining glomerular lesions. No significant differences were detected in interstitial lesions, although albuminuric patients showed greater severity of IFTA lesions and higher infiltration in areas without IFTA, both of which are assigned a higher score in the Tervaert score classification [5] (Table 2 and Figures 2–4).

Note that no significant differences were found between albuminuric and nonalbuminuric subjects in variables such as BMI, degree of blood pressure control, or lipid parameters. Although albuminuric patients had higher HbA1c over the previous year, this difference did not reach statistical significance as HbA1c levels were practically identical across the two groups in the two preceding years. Neither were any significant differences found in associated comorbidities or treatment received, with the exception that albuminuric patients received a higher percentage of insulin and metformin (*p* = 0.031 in both cases). It is striking that no patient in the albuminuric group had diabetic retinopathy (Table 1).

These data suggest that patients without albuminuria already have DN-associated histologic lesions despite the absence of clinical manifestations of the disease (microalbuminuria). Particularly noteworthy is that normoalbuminuric patients presented a similar degree of mild mesangial expansion (41.2% vs. 41.7%) and thickening of GBM to those with diabetes (84% vs. 86.5%); moreover, in some glomeruli, they showed severe mesangial expansion, although to a lesser degree than in albuminuric subjects, even though the median course of diabetes in nonalbuminuric patients was 90.5 months (interquartile range: 63–109.5).

## 4. Discussion

The first finding of our study is that a significant percentage of patients not displaying early clinical signs of DN such as microalbuminuria already show moderate to severe DN-related histologic involvement, although there were no significant differences in comorbidities and renal progression factors compared with the albuminuria patient group. These findings are suggestive of early renal involvement in nonalbuminuric T2DM patients, also supported by the fact that in our sample, no patient had diabetic retinopathy (nor in cases with albuminuria). These findings gain further importance considering that histologic studies cannot be performed in patients with early DN expression.

The prognostic implication of Tervaert classification scores [5] has been well established since An et al.'s work [10], supporting the predictive value of the previously described pathological classification of DN, especially for interstitial lesions. In most histologic studies performed in DN patients, the samples included predominantly grades IIb and IV and high proteinuria levels [10, 11] and thus had greater renal compromise than those analyzed in our study. Similarly, other studies analyzing kidney biopsies from DN patients also found considerable changes in arteriosclerosis [12].

In one of the few published studies including a large sample of patients in initial DN stages, Quinn et al. analyzed the histologic material of nephrectomy [13], evaluating 859 samples from partial or total nephrectomies from kidney neoplasia (304 (35.4%) patients with T2DM). Their findings demonstrate that including renal histologic variables with baseline eGFR contributes to a more precise estimation of kidney function decline, especially in the early stages of DN. The degree of histologic damage was highly variable in patients with CKD stages 1–2, and some individuals displayed relatively severe structural damage despite preserved eGFR. These findings were a high degree of fibrosis, tubular atrophy, and glomerulosclerosis.

On the other hand, our findings show clinical and pathological dissociation, further supporting recent controversy over the emerging concept of “DN without proteinuria” [14], which complicates the interpretation of histologic data in patients with different degrees of DN. Findings point toward the existence of two phenotypes of renal involvement in T2DM (with or without proteinuria), of which the latter can progress to ESKD without a significant degree of proteinuria [15].

The second interesting finding of our study was the high prevalence of arteriolar hyalinosis (66.7% in albuminuric and 56.3% in nonalbuminuric patients). Quinn et al. also reported a high degree of arterial hyalinosis, at 32.7%, although without distinguishing between diabetic and nondiabetic patients [13]. This has also been demonstrated in kidney biopsies from DN-affected patients, where 85% displayed arteriolar hyalinosis and 60% arteriolosclerosis [16]; this condition can also affect the arteries, with a preponderant role in glomerular hemodynamic changes. The high prevalence of these histologic alterations may have considerable implications for the renal adaptive mechanisms involved in glomerular hemodynamics, since arterioles with hyalinosis may have hampered mechanisms of vasoconstriction and vasodilation through which they exert their role in intraglomerular HTN, which could affect current nephroprotective treatments in T2DM such as renin-angiotensin-aldosterone system (RAAS) blockers and sodium/glucose cotransporter 2 inhibitors (SGLT2i) [17, 18].

This may partially account for the variability of response to nephroprotective treatments in T2DM. In the RENAAL (Reduction in End Points in Noninsulin-Dependent Diabetes Mellitus with the Angiotensin II Antagonist Losartan) study, the albuminuria reduction rate at 6 months showed that 41.6% of patients experienced no reduction in albuminuria; in 25.9%, albuminuria was lowered by 0–30%, and 32.3% saw an albuminuria decrease of over 30% [19]. Kraus et al. analyzed the initial eGFR dip after SGLT2i treatment in the EMPA-REG OUTCOME study [20]. They showed that in patients treated with empagliflozin, 31% showed no eGFR decline; in 41%, it dropped between 0 and 10%, and in 28%, the dip was between 10 and 30%. Only 1.4% showed an eGFR decline greater than 30%. These different responses to nephroprotective treatments may be due to multiple factors, including the extent of arterial hyalinosis in afferent and efferent arterioles. The high prevalence of these histologic alterations may have important implications for the renal adaptive mechanisms involved in glomerular hemodynamics.

Thirdly, our findings add weight to the importance of early and strict metabolic control in T2DM. Two post hoc analyses of the UKPDS study have shown that the glycemic legacy effects seen in T2DM are largely explained by historical HbA1c values, which have a greater impact on renal and cardiovascular outcomes than recent values; microvascular complications and mortality also increased in those patients who presented HbA1c >6.5% in the first year after diagnosis [21, 22]. Based on our findings and supported by these post hoc studies, we could suggest that early detection of diabetes and intensive glucose control from the time of diagnosis are essential for maximum reduction of long-term risk of glycemic complications.

The VERIFY study recently demonstrated that early intervention with combination therapy of vildagliptin plus metformin provides greater and more durable long-term benefits in newly diagnosed T2DM patients compared with metformin as monotherapy [23]. This result also supports that early and intense intervention can improve metabolic control in these patients. In this regard, the DECLARE study in T2DM patients with or at risk of atherosclerotic cardiovascular disease found that treatment with dapagliflozin was associated with a 24% reduction in the renal endpoint (hazard ratio, 0.76; 95% CI, 0.67–0.87). These findings demonstrate the effect of an SGLT2i in earlier stages of renal disease since in this study, 65% of patients showed no expression of any renal markers (eGFR < 60 mL/min/1.73 m^2^, microalbuminuria or macroalbuminuria: 93% showed eGFR > 60 mL/min/1.73 m^2^ and 69.1% showed no albuminuria) and 59.4% of patients had no previous cardiovascular disease [24, 25]. Interestingly, the use of SGLT2i seems to prevent and reduce diabetic kidney disease progression compared to placebo in this large and diverse population study in T2DM patients with or without established atherosclerotic cardiovascular disease, most of whom had preserved renal function. Although the possible mechanisms of benefit were multiple and incompletely understood, in most cases, this benefit has been independently associated with glycemic control. Based on current evidence, KDIGO guidelines recommend that glycemic management for T2DM and CKD patients should include lifestyle therapy and first-line treatment with metformin and an SGLT2i [3].

Our study has several limitations, such as the low number of samples used for analysis, the presence of tubulointerstitial lesions inherent to acute tubular necrosis or nephrotoxic agent history in some histologic samples, and the lack of additional pathologists to avoid single observer bias. Furthermore, there were just nine autopsies in patients with albuminuria and a slight imbalance between age in the groups and other variables. That HbA1c analysis was carried out in the previous two years could be considered a limitation of the study, as could interpretation of these results without considering metabolic control history. Nonetheless, the exhaustive histologic analysis applying updated scores and the high number of glomeruli analyzed in each sample allowed us to obtain significant results.

## 5. Conclusion

Our data suggest that renal involvement in T2DM patients is present in earlier stages of the disease (patients without albuminuria), highlighting the importance of early metabolic control as well as control of other risk factors which may influence DN development. Large sample size studies are warranted to confirm these data and to further study new therapeutic options such as SGLT2i and/or glucagon-like peptide-1 receptor agonists which show great promise in DN prevention. These findings can serve as a basis to support early and strict metabolic control in patients with DM and the use of drugs with demonstrated renal and cardiovascular benefit in the treatment of this disease, all of which will undoubtedly improve prognosis in affected patients.

## Figures and Tables

**Table 1:** General patient characteristics: most recent clinical and analytical data (median and interquartile range, percentage).

Variable	Type 2 diabetic patients		Control (*n* = 4)	*p* value alb vs no alb	*p* value control vs T2DM
	Albuminuric (*n* = 9)	Nonalbuminuric (*n* = 12)	Control (*n* = 4)		
Age (years)	75 [64–90.5]	77.5 [68.25–83.75]	81 [58.5–82.5]	0.890	0.803
Male sex (%)	77.8	75.0	75.0	1	1
Diabetes mellitus vintage (mo)	108 [56–124.5]	90.5 [63–109.5]	NA	0.422	NA
Weight (kg)	68.5 [63–NA]	78.5 [69.5–102.25]	75 [47–NA]	0.198	0.885
Height (cm)	161 [156–168.5]	165 [159.75–171.25]	160 [159–NA]	0.500	0.875
BMI (kg/m^2^)	31.8 [27.45–36.4]	30.35 [26.7–34.6]	29.7 [18.7–NA]	0.559	0.559
Systolic BP (mmHg)	130 [87–148.5]	110 [100–138]	124.5 [117–140.25]	0.519	0.477
Diastolic BP (mmHg)	65 [55–72]	61 [54–72]	63.5 [51–75.25]	0.815	0.852
Mean blood pressure (mmHg)	90.33 [65.67–95.5]	72.33 [69.25–90.75]	NA	0.754	NA
Last albuminuria (mg/g Cr)	223.33 [153–393.7]	10.53 [3.08–17.69]	2.6 [2.6–2.6]	<0.001	0.200
Albuminuria 1 year before (mg/g Cr)	220.22 [82–294–36]	7.33 [3.34–14.22]	NA	0.001	NA
Albuminuria 2 years before (mg/g Cr)	217.02 [86.40 332.64]	6.72 [3.64–19.45]	NA	<0.001	NA
Last serum creatinine (mg/dL)	1.89 [1.03–4]	1.02 [0.72–2.01]	0.68 [0.41–1.73]	0.032	0.068
Serum creatinine 1 year before (mg/dL)	1.49 [0.96–2.03]	1 [0.82–1.49]	NA	0.111	NA
Serum creatinine 2 years before (mg/dL)	1.16 [0.96–1.81]	0.91 [0.73–1.14]	NA	0.058	NA
eGFR <60 mL/min/1.73 m^2^ (%)	83.3	31.3	25.0	0.056	0.594
Last eGFR (mL/min/1.73 m^2^)	22.96 [15.87–65.08]	70.84 [45.01–85.65]	85.55 [43.13–96.4]	0.037	0.231
eGFR 1 year before (mL/min/1.73 m^2^)	44.26 [31.95–68.05]	74.99 [51.13–85.87]	NA	0.095	NA
eGFR 2 years before (mL/min/1.73 m^2^)	50.07 [35–77.96]	82.47 [59.12–88.78]	NA	0.095	NA
Serum albumin (day/dL)	3.3 [2.75–4.15]	3.4 [3.2–3.85]	3.2 [3–NA]	0.250	0.569
Cholesterol (mg/dL)	142 [122–173]	169.5 [132.5–176.75]	151 [122.5–173.5]	0.276	0.695
Triglycerides (mg/dL)	100 [72–147.5]	138 [70–156]	70 [48–NA]	0.599	0.172
Glycemia (mg/dL)	143 [96–182.5]	119.5 [107.5–161]	102 [89–111.25]	0.760	0.031
Last HbA1c (%)	7.56 [6.53–9.43]	6.75 [6.13–7.55]	NA	0.238	NA
HbA1c 1 year before (%)	7 [6.33–8.9]	6.9 [6.4–7.35]	NA	0.734	NA
HbA1c 2 years before (%)	6.6 [6.1–8.4]	6.65 [6.4–7.35]	NA	0.917	NA
HbA1c <7.5% (%)	33.3	33.3	NA	1.000	1.000
Diabetic retinopathy	11.1	0	NA	0.429	1.000

Variables are expressed as median and interquartile ranges. eGFR <60 mL/min/1.73 m^2^, male sex, retinopathy, and HbA1c <7.5% in %. NA: not available (not calculable).

**Table 2:** Renal histologic findings in necropsies of T2DM (albuminuric and nonalbuminuric) patients and nondiabetic controls.

	Albuminuric (*n* = 9)	Nonalbuminuric (*n* = 12)	Controls [4]	*p* value alb vs no alb	*p* value control vs T2DM
Total number of glomeruli in the sample	113 [94–228.5]	159.5 [120.75–213.5]	129.5 [82.25–186.5]	0.207	0.452
GBM thickening, *n*	100 [59–130]	111 [30.25–171.25]	0 [0–12.75]	0.846	<0.001
GBM thickening (%)	84 [43.5–92.5]	86.5 [21–93.75]	0 [0–12.75]	0.890	<0.001
Mild mesangial expansion, *n*	100 [59–130]	111 [30.25–171.25]	0 [0–12.75]	0.846	<0.001
Mild mesangial expansion (%)	41.2 [26–107.5]	47.1 [11.25–134.25]	10 [0–23.75]	0.890	0.015
Severe mesangial expansion (%)	11 [0.5–42.5]	3 [0–67.25]	0 [0–0]	0.637	0.015
Advanced glomerular sclerosis (>50%) (*n*)	8 [5.5–21.5]	8 [4.25–15.75]	1 [0–30.5]	0.677	0.113
Nodular sclerosis (KW) (yes/no) (%)	66.7	18.8	0	0.031	0.260
Arteriolar hyalinosis (yes/no) (%)	66.7	56.3	0	0.691	0.017
Presence of arteriosclerosis (%)	88.9	91.7	75.0	0.841	0.408
Presence of interstitial lesions (%)	28.6	18.8	0	0.621	0.539
IFTA (%)	10 [5–27.5]	5 [1–22.5]	1 [0–3.75]	0.456	0.006
IFTA					
(i) No IFTA	12.5	23.1	72.0		
(ii) <25%	62.5	53.8	25.0		
(iii) 25–50%	12.5	23.1	0		
(iv) >50%	12.5	0	0	0.653	0.037
Inflammation					
(i) Absent	11.1	22.2	75.0		
(ii) Infiltration only related to IFTA	55.6	77.8	0		
(iii) Infiltration in areas without IFTA	22.2	11.1	25.0	1.000	0.023

GBM, glomerular basement membrane; KW, Kimmelstiel-Wilson; IFTA, interstitial fibrosis and tubular atrophy.

## Data Availability

Data are available upon request.

## Supplementary Materials

Supplementary Materials The supplementary table shows the general characteristics of the patients. Thus, the last clinical and analytical data known are presented in median values, interquartile range, and percentage.
