# Supplementary material for: Renal Histologic Findings in Necropsies of Type 2 Diabetes Mellitus Patients
Source: J Diabetes Res. 2022 Sep 6;2022:3893853. doi: 10.1155/2022/3893853 (PMC9470369; doi:10.1155/2022/3893853)
Supplement: Supplementary Materials — The supplementary table shows the general characteristics of the patients. Thus, the last clinical and analytical data known are presented in median values, interquartile range, and percentage. [file 3893853.f1.docx]

**Supplementary table.** General characteristics of the patients: Last clinical and analytical data known (median and interquartile range, and percentage).

| ***Variable*** | **Type 2 DM patients** | | **Control (n=4)** |  |  |
| --- | --- | --- | --- | --- | --- |
|  | **Albuminuric (n=9) %** | **Non-albuminuric (n=12) %** | **Control (n=4)**  **%** | **P-value**  **Alb vs No Alb** | **P-value**  **Control Vs T2DM** |
| T2DM duration> 10 y. % | 77.8 | 83.3 | NA | 1.000 | 1.000 |
| Obesity, % | 80 | 45 | 50 | 1.000 | 1.000 |
| History of CVD, % | 55.6 | 62.7 | 75 | 1.000 | 0.627 |
| History of coronary heart disease, % | 22.2 | 25.0 | 50 | 1.000 | 0.234 |
| History of stroke/TIA, % | 11.1 | 25 | 25 | 0.621 | 1.000 |
| History of peripheral vascular disease, % | 22.2 | 6.3 | 0 | 0.530 | 1.000 |
| History of heart failure, % | 25.0 | 18.8 | 50 | 1.000 | 0.166 |
| Treatment with ASA, % | 22.2 | 62.5 | 50 | 0.097 | 1.000 |
| Treatment with RAAS blockers, % | 55.6 | 50 | 100; 0 | 1.000 | 1.000 |
| Sulfonylurea, % | 0 | 12.5 | - | 0.520 | NA |
| Metformin, % | 66.7 | 18.8 | - | 0.031 | NA |
| Treatment with iDPP4, % | 55.6 | 25 | - | 0.200 | NA |
| Treatment with repaglinide, % | 22.2 | 18.8 | - | 1.000 | NA |
| Insulin therapy, % | 66.7 | 18.8 | - | 0.031 | NA |

T2DM, Type 2 diabetes mellitus; CVD, cardiovascular disease; TIA; transitory ischemic accident; ASA, acetylsalicylic acid; RAAS, renin-angiotensin-aldosterone system; iDPP4, Inhibitors of dipeptidyl peptidase 4.
